# Genome-wide association study of type 2 diabetes in Africa

**DOI:** 10.1007/s00125-019-4880-7

**Published:** 2019-05-02

**Authors:** Ji Chen, Meng Sun, Adebowale Adeyemo, Fraser Pirie, Tommy Carstensen, Cristina Pomilla, Ayo P. Doumatey, Guanjie Chen, Elizabeth H. Young, Manjinder Sandhu, Andrew P. Morris, Inês Barroso, Mark I. McCarthy, Anubha Mahajan, Eleanor Wheeler, Charles N. Rotimi, Ayesha A. Motala

**Affiliations:** 1 Wellcome Sanger Institute, Hinxton, Cambridge, UK; 2 Wellcome Centre for Human Genetics, University of Oxford, Roosevelt Drive, Oxford, OX3 7BN UK; 3 Center for Research on Genomics and Global Heath, National Human Genome Research Institute, National Institute of Health, Bethesda, MD USA; 4 Department of Diabetes and Endocrinology, University of KwaZulu-Natal, Durban, 4013 South Africa; 5 Department of Medicine, University of Cambridge, Cambridge, UK; 6 Department of Biostatistics, University of Liverpool, Liverpool, UK; 7 MRC Epidemiology Unit, University of Cambridge School of Clinical Medicine, Institute of Metabolic Science, Cambridge Biomedical Campus, Cambridge, CB2 0QQ UK; 8 Oxford Centre for Diabetes, Endocrinology and Metabolism, University of Oxford, Oxford, UK; 9 Oxford NIHR Biomedical Research Centre, Oxford, UK

**Keywords:** Africa, Established loci, Fine-mapping, Genome-wide association study, Type 2 diabetes

## Abstract

**Aims/hypothesis:**

Genome-wide association studies (GWAS) for type 2 diabetes have uncovered >400 risk loci, primarily in populations of European and Asian ancestry. Here, we aimed to discover additional type 2 diabetes risk loci (including African-specific variants) and fine-map association signals by performing genetic analysis in African populations.

**Methods:**

We conducted two type 2 diabetes genome-wide association studies in 4347 Africans from South Africa, Nigeria, Ghana and Kenya and meta-analysed both studies together. Likely causal variants were identified using fine-mapping approaches.

**Results:**

The most significantly associated variants mapped to the widely replicated type 2 diabetes risk locus near *TCF7L2* (*p* = 5.3 × 10^−13^). Fine-mapping of the *TCF7L2* locus suggested one type 2 diabetes association signal shared between Europeans and Africans (indexed by rs7903146) and a distinct African-specific signal (indexed by rs17746147). We also detected one novel signal, rs73284431, near *AGMO* (*p* = 5.2 × 10^−9^, minor allele frequency [MAF] = 0.095; monomorphic in most non-African populations), distinct from previously reported signals in the region. In analyses focused on 100 published type 2 diabetes risk loci, we identified 21 with shared causal variants in African and non-African populations.

**Conclusions/interpretation:**

These results demonstrate the value of performing GWAS in Africans, provide a resource to larger consortia for further discovery and fine-mapping and indicate that additional large-scale efforts in Africa are warranted to gain further insight in to the genetic architecture of type 2 diabetes.

**Electronic supplementary material:**

The online version of this article (10.1007/s00125-019-4880-7) contains peer-reviewed but unedited supplementary material, which is available to authorised users.

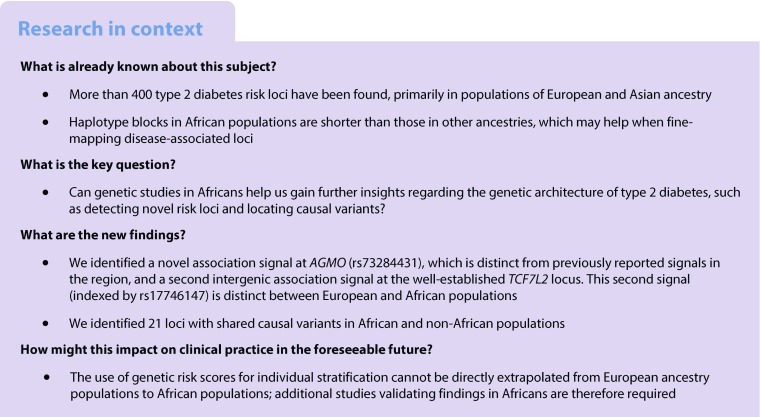



## Introduction

Type 2 diabetes is a major and growing public health problem, with Africa being the region with the fastest growing prevalence [1–3]. In addition to lifestyle, genetic factors play a major role in susceptibility to type 2 diabetes. To date, type 2 diabetes genome-wide association studies (GWAS) have uncovered over 400 risk signals, primarily in populations of European [4, 5] and Asian [6–8] ancestry, with more limited efforts in Hispanics/Latinos [9–11] and African-Americans [12, 13]. Thus far, type 2 diabetes genetic studies in populations from Africa, which are genetically and environmentally diverse, have focused on the replication of established loci [14].

Here, we conducted a meta-analysis of type 2 diabetes in up to 4347 African participants to identify genetic risk factors associated with type 2 diabetes in Africans, evaluate previously reported loci and utilise the finer-grained linkage disequilibrium (LD) pattern of African populations to fine-map-associated loci.

## Methods

### Study participants

South African Zulu individuals were type 2 diabetes cases and controls from two studies: the Durban Diabetes Study (DDS) and the Durban Diabetes Case Control study (DCC). DDS was a population-based cross-sectional study of non-pregnant urban black African adults of Zulu descent, aged >18 years, residing in Durban, South Africa [15, 16]. Further details are provided in the electronic supplementary material (ESM) Methods. Additional type 2 diabetes cases from the same ethnic group and locality were obtained from the DCC, which included individuals with type 2 diabetes attending a diabetes clinic. Type 2 diabetes was defined using WHO criteria [15, 16]. The combined type 2 diabetes cases and controls from DDS and DCC were aggregated into a single Zulu study.

The Africa America Diabetes Mellitus (AADM) study comprised individuals from sub-Saharan Africa, enrolled from university medical centres in Nigeria, Ghana and Kenya. A person with type 2 diabetes was identified using ADA criteria or if he or she was receiving treatment for type 2 diabetes. Probable cases of type 1 diabetes were excluded and controls had no evidence of diabetes based on fasting/2 h glucose or symptoms of suggestive diabetes [14]. The characteristics of the Zulu and AADM participants are shown in ESM Table 1.

### Genotyping, quality control and imputation

In total, 2707 African individuals of Zulu descent (2003 women and 704 men) were genotyped using the Illumina Multi-Ethnic Genotyping Array (Illumina, Illumina Way, San Diego, CA, USA). Following sample and variant quality control (QC) (ESM Methods), there were 2578 samples with genotype and phenotype information (1602 cases and 976 controls) and 1,434,868 variants (1,395,345 autosomal and 39,523 on the X chromosome).

The AADM samples were genotyped on the Affymetrix Axiom PANAFR SNP array as described previously [14]. After QC (ESM Methods), there were 1031 cases and 738 controls and 2,141,465 variants (2,080,378 autosomal and 61,087 on the X chromosome) in AADM.

All samples were imputed to a merged panel of 1000 Genomes phase 3 [17] and African samples using IMPUTE2 [18] (Zulu) or positional Burrows–Wheeler transform (PBWT) [19] using the Sanger imputation server (AADM) (ESM Methods). We retained all imputed SNPs with MAF > 0.01 and imputation information score > 0.4 that were also in a newer version of the imputation panel.

### Association analysis

In Zulu samples, association with type 2 diabetes was performed for each variant based on the imputation dosage using a linear mixed model that accounts for the presence of related individuals and any population structure implemented in genome-wide efficient mixed-model association (GEMMA) [20], adjusting for age, sex and BMI. The kinship matrix was estimated from directly genotyped autosomal variants with MAF > 0.01. In AADM, association with type 2 diabetes was performed for each variant based on the imputation dosage using an additive logistic regression model implemented in SNPTEST v2.5.2 [21] (Oxford University, Oxford, UK www.well.ox.ac.uk/~gav/resources/snptest_v2.5.2_linux_x86_64_dynamic.tgz) adjusting for age, sex, BMI and the first three principal components (PCs) to account for population structure. The number of PCs adjusted for in AADM was determined by testing for the number of PCs using the minimum average partial test [22]. The first three PCs were determined to be significant and thus were adjusted for in the association analyses.

### Meta-analysis, signal selection and fine-mapping

Meta-analysis of the Zulu and AADM summary statistics for shared variants was performed using a fixed-effects meta-analysis (weighted for effective sample size) in METAL [23] and we applied double genomic-control correction (ESM Methods). To identify distinct signals of association, we performed approximate conditional analyses using the joint model implemented in genome-wide complex trait analysis (GCTA) [24, 25] and variants with *p* < 2.5 × 10^−8^ in the joint model were selected as signals with genome-wide significance [26]. To estimate meta-analysis ORs, we also performed an inverse-variance-weighted meta-analysis using an approximation of the allelic log_*e*_OR and variance from the linear model in the Zulu samples [27], and the log_*e*_OR estimates obtained directly from SNPTEST for the AADM samples.

We used FINEMAP [28] (C. Benner, University of Helsinki, Finland www.christianbenner.com/finemap_v1.1_x86_64.tgz) to identify likely causal variants within 500 kb either side of the most significant variant at the loci *TCF7L2* and *AGMO* in the African meta-analysis and in Europeans [5] (ESM Methods).

### Comparison with established loci

We used ‘direct’ (same lead variant with *p* < 0.05 and directionally consistent) and ‘local’ (locus-level) detection to explore the extent to which existing GWAS signals (almost all from non-African samples) were detected in the African GWAS (ESM Methods, ESM Table 2). We used two complementary approaches to test for enrichment of signals detected using the ‘direct’ approach: (1) a binomial test taking significance (*p* < 0.05) and direction of effect into account; and (2) enrichment of directly detected variants, accounting for the properties of the variants in GARFIELD [29] (ESM Methods). For loci demonstrating ‘direct’ and/or ‘local’ detection between our African data and existing GWAS signals (ESM Table 2), we performed co-localisation analyses implemented in the R package ‘coloc’ [30] using summary statistics from the largest available European type 2 diabetes GWAS at the time of analysis [5] (default prior for causal variant sharing set to 0.5) (ESM Methods). Using the weighted allele frequencies and sample sizes from the African meta-analysis and previously reported effect sizes, we estimated the power to detect established variants at the significance thresholds *p* < 0.05 and *p* < 2.5 × 10^−8^ using R version 3.3.0 [31] (ESM Table 2).

We also performed genetic risk score (GRS) analyses to harvest association information from multiple variants. GRSs were calculated as the total number of risk alleles in subsets of the 102 variants at established loci from existing GWAS studies of type 2 diabetes (published before May 2018), primarily in populations of European and Asian ancestry (ESM Table 2).

### Testing association of *INS-*variable number tandem repeat with type 2 diabetes

We used the haplotypic information for *INS-*variable number tandem repeat **(**VNTR) generated in African-descent individuals by Stead et al (2003) [32] to impute *INS-*VNTR lineages in the Zulu and AADM samples and perform a meta-analysis (ESM Methods). Conditional analysis was performed to detect distinct association signals by inclusion of dosages of the lead type 2 diabetes variants as covariates in the regression model.

### Ethics statements

Ethical approval was obtained for each participating cohort: the Institutional Review Board for each AADM participating institution and the Biomedical Research Ethics Committee of the University of KwaZulu-Natal for DCC (BF078/08) and DDS (BF030/12). DDS also had UK National Research Ethics Service approval (reference: 14/WM/1061). Written informed consent was obtained from all participants and the study was conducted in accordance with the principles of the Declaration of Helsinki.

## Results

**Fig. 1:**
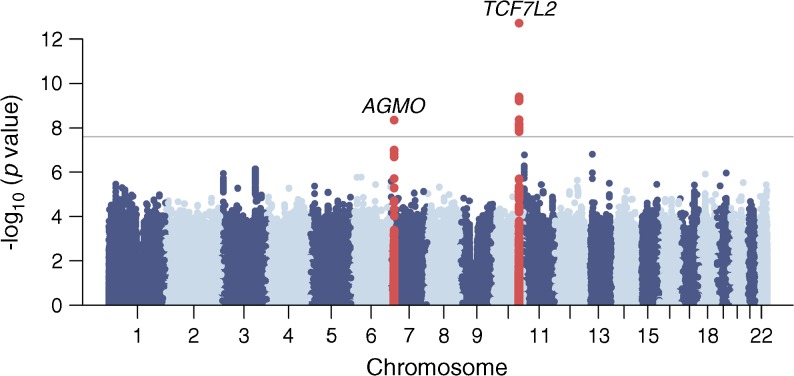
Manhattan plot of the type 2 diabetes meta-analysis results. The horizontal grey line corresponds to *p*=2.5×10^−8^ and loci reaching that significance threshold (variants within 500 kb distance of those with *p*<2.5×10^−8^) are shown in red. Gene labels correspond to the nearest/most biologically plausible gene

A total of 12,148,595 variants (genotyped or imputed) overlapping the Zulu and AADM samples were included in the meta-analysis of type 2 diabetes. We identified two genome-wide-significant (*p* < 2.5 × 10^−8^) [26] association signals (Table 1, Fig. 1). A further 37 distinct signals were detected at 2.5 × 10^−8^ ≤ *p* < 1 × 10^−5^ (ESM Table 3).

**Table 1:** Type 2 diabetes susceptibility loci with genome-wide significance in combined Zulu and AADM meta-analysis

SNP	Chr.	Position (bp)	Alleles^a^		Locus/nearest gene(s)	Zulu GWAS			AADM GWAS			Meta-analysis			
			Risk	Other		RAF	*p* value	OR (95% CI)	RAF	*p* value	OR (95% CI)	Weighted mean RAF	*p* value	OR (95% CI)	Heterogeneity *p* value
rs7903146	10	114,758,349	T	C	*TCF7L2*	0.381	7.9 × 10^−7^	1.25 (1.15, 1.37)	0.319	1.5 × 10^−8^	1.58 (1.34, 1.85)	0.356	5.3 × 10^−13^	1.32 (1.22, 1.43)	0.26
rs73284431	7	15,434,230	G	C	*AGMO*	0.924	2.1 × 10^−8^	1.59 (1.35, 1.87)	0.878	0.011	1.31 (1.06, 1.62)	0.905	5.2 × 10^−9^	1.48 (1.30, 1.69)	0.093

Genome-wide significance, *p*<2.5×10^−8^

^a^Alleles are aligned to the forward strand of NCBI Build 37

Chr., chromosome; RAF, risk allele frequency; heterogeneity *p* value: *p* value from Cochran’s *Q* test for heterogeneity

The most significant signal for type 2 diabetes association, rs7903146 at *TCF7L2* (*p* = 5.3 × 10^−13^), has been widely reported in other ethnic groups [9, 13, 33, 34]. The second signal, rs73284431, (*p* = 5.2 × 10^−9^, risk allele frequency = 0.093, specific to individuals of African descent) maps to an intron of *AGMO*, 491 kb upstream of the *DGKB* transcription start site. The region is already known to harbour two independent GWAS signals for type 2 diabetes [5] and fasting glucose [35, 36] (denoted by rs10238625 and rs10276674, 132 kb apart and >379 kb from rs73284431), both of them distinct from our lead variant based on exact conditional analyses (ESM Table 4).

Despite not reaching genome-wide significance, the association with rs12277475 (*p* = 2.0 × 10^−7^, ESM Table 3) near the *INS* (insulin) gene was of interest given previous reports of a significant African-American type 2 diabetes association signal at rs3842770 [37], 28 kb away. The two lead variants are not in LD (*r*^2^ ~ 0.03) in 2959 African samples from the merged imputation reference panel [13], and reciprocal exact conditional analyses confirmed our signal was not being driven by the reported African-American association signal (ESM Table 5). In our data, rs3842770 showed a more modest association with type 2 diabetes (*p* = 0.0020 in the joint meta-analysis) than rs12277475. The rs12277475 association was also not driven by the previously reported East Asian type 2 diabetes signal (rs7107784) in this region [7] (ESM Table 5).

In type 1 diabetes, robust associations with variants at *INS-IGF2* have been localised to the *INS*-VNTR mini-satellite within the *INS* gene promoter [38, 39]; in Europeans, an association between VNTR class III alleles and type 2 diabetes predisposition has been reported in historical candidate gene studies [40, 41]. To explore the relationship between the rs12277475 signal and VNTR variation, we imputed *INS*-VNTR genotypes using flanking SNP haplotypes (see Methods and ESM Methods). Of the nine distinct lineages with MAF > 0.01 detected in both Zulu and AADM, the strongest associations were observed for lineage W (*p* = 0.0001, OR 1.24, ESM Table 6) and lineage K (*p* = 0.0057, OR 0.85, ESM Table 6). Type 2 diabetes association results for lineages W and K and lead variant rs12277475 were largely unchanged in reciprocal conditional analyses (ESM Tables 7 and 8 [Zulu and AADM, respectively]). We conclude that rs12277475 is likely to represent a novel type 2 diabetes association in this region, independent of previous genome-wide significant associations detected in the close vicinity, and is not acting through the VNTR.

### Fine-mapping of *TCF7L2* and *AGMO*

Fine-mapping of *TCF7L2* and *AGMO* identified the most significant variants from the meta-analysis, rs7903146 and rs73284431, as the most likely causal variants with posterior probabilities of association of 0.996 and 0.828, respectively (ESM Table 9). These were the only SNPs in the top configuration of causal variants from FINEMAP (ESM Figs. 1 and 2). At *TCF7L2,* a second plausible causal variant, rs17746147 (*r*^2^ = 0.009 with rs7903146 estimated from the African samples in the merged panel), with posterior probability of association of 0.295, was contained in the second most likely configuration of causal variants (ESM Table 9, ESM Fig. 1). The 99% credible intervals and corresponding results in Europeans are presented in ESM Table 9. Fine-mapping results were comparable when using the stepwise approximate Bayes factor approach [42] (data not shown).

### Detection of established loci

We explored the extent to which previously reported type 2 diabetes association signals could be detected in African-descent individuals. Based on the previously reported effect sizes and the effect allele frequency and sample size from our African meta-analysis, we had sufficient power (80%) to detect three signals (*TCF7L2*, *DNER* and *SRR*) at genome-wide significance (*p* < 2.5 × 10^−8^) (ESM Table 2). Only the *TCF7L2* variant reached genome-wide significance in our study, whereas both variants in *DNER* (rs1861612) and *SRR* (rs391300), originally discovered in Pima Indians and East Asians, respectively, had *p* > 0.1 (ESM Table 2).

So far, five African-American type 2 diabetes-associated signals have been reported, three of which (two in *KCNQ1* and one in *HMGA2*) were first reported in Europeans and two (*INS-IGF2* and *HLA-B*) were first reported in African-Americans (ESM Table 2) [13]. In our meta-analysis, we detected only a nominal association for the African-American *INS-IGF2* signal (rs3842770, *p* = 0.0020, ESM Table 2). However, we identified another signal at this locus (rs12277475, *p* = 2.0 × 10^−7^, ESM Table 3) that is independent of the African-American signal (rs3842770), and this signal co-localises with association in Europeans (posterior probability H4 = 1, ESM Table 10).

In ‘direct’ analyses (same lead variant with *p* < 0.05 and directionally consistent in the African meta-analysis), we detected 12 of 100 lead variants (ESM Table 2) at established type 2 diabetes loci first reported in non-African ancestry individuals, significantly more than expected by chance (binomial test of enrichment *p* = 8.14 × 10^−6^, GARFIELD enrichment OR [95% CI] 3.07 (1.50, 6.28), *p* = 0.002) (ESM Methods). In addition, detection of ‘local’ signals (at least one variant within the 200 kb region flanking the previously reported index variant [100 kb either side] reaching nominal significance [*p* < 0.05] after correcting for the effective number of independent tests) identified 11 type 2 diabetes loci, two of them overlapping signals also detected with the direct approach (ESM Table 2). Genetic co-localisation analyses suggested that African and non-African populations share the same causal variants at all 21 loci showing direct or local detection in our data (posterior probability H4 > 0.8, ESM Table 10).

**Fig. 2:**
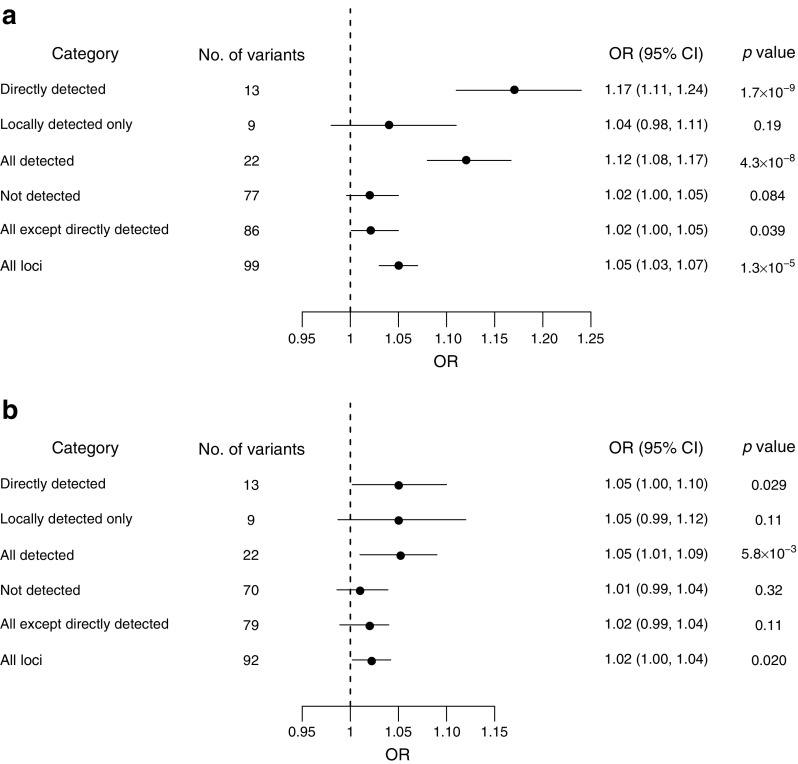
(**a**) Associations between the GRSs constructed from the subsets of established type 2 diabetes variants and type 2 diabetes in the Zulu samples. (**b**) Associations between the GRSs constructed from the subsets of established type 2 diabetes variants and type 2 diabetes in the AADM samples. Please see ESM Table 2 for details of categories

We constructed GRSs by combining subsets of 102 previously established loci (including those first reported in African-ancestry individuals) and tested for association with type 2 diabetes (ESM Table 2). A GRS constructed from these variants showed significant association with type 2 diabetes in the Zulu (OR 1.05 per risk allele, *p* = 1.3 × 10^−5^, Fig. 2a) and AADM samples (OR 1.02 per risk allele, *p* = 0.020, Fig. 2b), an association driven primarily by the 13 directly detected variants (Zulu, OR 1.17, *p* = 1.7 × 10^−9^; AADM, OR 1.05, *p* = 0.029). GRSs based on the variants detected by only the local approach or the variants not detected by either approach were not significantly associated with type 2 diabetes in African samples (*p* = 0.19 and 0.084, respectively in the Zulu; *p* = 0.11 and 0.32, respectively in AADM) (Fig. 2). These results show there is a shared genetic contribution to type 2 diabetes at established loci directly detected in Africans.

## Discussion

In a meta-analysis of type 2 diabetes from two African populations, we replicated the widely reported association at *TCF7L2* (rs7903146) and identified a novel association signal at *AGMO* (rs73284431) that is distinct from previously reported signals in the region.

Using direct and local detection, we showed the transferability of 21 established type 2 diabetes signals discovered in non-African ancestry populations to Africans and that causal variants at those loci were shared across ancestries. For example, the lead SNP at *TCF7L2*, rs7903146, shared between African and European ancestries, has been refined as the causal variant by examining LD blocks of West African, Danish, Icelandic and American-African populations [43, 44]. Although the mechanisms through which *TCF7L2* variation increases type 2 diabetes risk are largely unknown, recent evidence implicates altered incretin signalling [45].

We also found evidence for ancestry-specific signals, such as the second intergenic association signal at *TCF7L2* that is distinct between European and African populations (indexed by rs17746147). In addition, although not reaching genome-wide significance, we detected an association at the *INS-IGF2* locus (rs12277475) that appears to be distinct from the previously reported signals in African-Americans and East Asians in the region. We found no evidence of association between the lead variant and VNTR variation in our study, suggesting that the association at *INS-IGF2* in our data is not acting through the VNTR.

In summary, our findings highlight the importance of diverse ancestries for uncovering novel biology. Larger African meta-analyses are warranted to gain further insight on the genetic architecture of type 2 diabetes.

## Electronic supplementary material

ESM (PDF 556 kb)

ESM Tables (XLSX 68 kb)

## Data Availability

Summary statistics from the meta-analysis will be available to download from the EBI GWAS Catalog (www.ebi.ac.uk/gwas/downloads/summary-statistics).

## Abbreviations

AADM: Africa America Diabetes Mellitus
DCC: Durban Diabetes Case Control study
DDS: Durban Diabetes Study
GRS: Genetic risk score
GWAS: Genome-wide association study
LD: Linkage disequilibrium
MAF: Minor allele frequency
NIHR: National Institute for Health Research
PC: Principal component
QC: Quality control
VNTR: Variable number tandem repeat
