# Supplementary material for: Genome-wide association study of type 2 diabetes in Africa
Source: Diabetologia. 2019 May 2;62(7):1204–11. doi: 10.1007/s00125-019-4880-7 (PMC6560001; doi:10.1007/s00125-019-4880-7)
Supplement: Supplementary file 1 — (PDF 556 kb) [file 125_2019_4880_MOESM1_ESM.pdf]

Genome-wide association study of Type 2 Diabetes in Africa

Electronic supplementary material

Table of Contents

ESM Methods 2
Study Participants 2
Genotyping, quality control and imputation 3
Meta-analysis, signal selection and fine-mapping 5
Comparison with established loci 6
Testing association of *INS*-VNTR with type 2 diabetes 8
References 9
ESM Figures 11
ESM Tables (see the separate file) 13

ESM Methods

Study Participants

Durban Diabetes Study (DDS)

The Durban Diabetes Study (DDS) was a population-based cross-sectional study, using multistage cluster sampling of non-pregnant urban black African individuals aged >18 years, of Zulu descent and residing in the city of Durban in KwaZulu-Natal (South Africa), conducted between November 2013 and December 2014. A detailed description of the survey design and procedures has been previously published [1, 2]. In brief, all consenting participants had anthropometric, demographic and biochemical measurements including a 75g OGTT using 1998 WHO criteria for disorders of glycaemia; whole blood was collected for DNA extraction. T2D cases were identified if they were ≥ 18 years old and had HbA1c > 6.5%, fasting plasma glucose ≥ 7.0 mmol/L, 2 hour plasma glucose ≥ 11.1 mmol/L, had been told by a doctor or health professional they had diabetes or were currently receiving insulin for diabetes. This analysis includes data on 1,104 participants (128 type 2 diabetes and 976 controls), on whom complete information was available.

Durban Diabetes Case Control study (DCC)

Subjects enrolled in the Durban Diabetes Case Control study (DCC) were all South African Zulu. All had a diagnosis of type 2 diabetes (based on WHO 1998 criteria) and were attending a diabetes clinic at either Inkosi Albert Luthuli Central Hospital or one of 3 peripheral clinics. Information relating to the diagnosis and treatment of diabetes, family history and lifestyle factors was obtained from each patient. Body weight was measured in light clothing on an electronic scale and blood pressure was measured in a seated position with an automated sphygmomanometer.

The cohort included 1,599 subjects with type 2 diabetes, of whom 1,214 (75.9%) were women. A family history of diabetes was present in 638 (40%) and of these, a maternal history was present more often (69.9%), than paternal (19.6%). The mean age was 56.4 ± 8.9 years, the mean age at diagnosis of diabetes was 49.8 ± 9 years and the mean duration of diabetes was 6.61 ± 6.74 years. The mean body mass index was 34.4 ± 7.1 kg/m^2 and hypertension was present in 1,338 (83.7%) subjects. The commonest macrovascular complication was cerebrovascular disease (3.7%) and the most frequently reported microvascular complication was retinopathy (23%).

DNA was extracted from EDTA-anticoagulated peripheral venous blood with a commercial kit (Nucleon BACC Genomic DNA Extraction Kits, GE Healthcare).

The Africa America Diabetes Mellitus (AADM)

The Africa America Diabetes Mellitus (AADM) study comprised individuals from sub-Saharan Africa (SSA), enrolled from university medical centres in Nigeria, Ghana, and Kenya. T2D was defined using the American Diabetes Association (ADA) criteria, or if an individual was receiving treatment for T2D. Probable cases of type 1 diabetes were excluded and controls had no suggestive evidence of diabetes based on fasting glucose/2hr glucose/symptoms of suggestive diabetes.

Genotyping, quality control and imputation

In total, 2,707 African individuals of Zulu descent (2,003 females and 704 males) were genotyped using the customized Illumina Multi-Ethnic Genotyping Array (MEGA) (Illumina, Illumina Way, San Diego, CA, US www.illumina.com/science/consortia/human-consortia/multi-ethnic-genotyping-consortium.html). Following genotyping, variants were called using Illumina's GenCall method with the default clustering file. All samples were

typed across 22 SNPs on the Sequenom® (Sequenom® Inc. California, USA) for sample quality control (QC genotypes). Samples were removed if they had genotype identity concordance with the QC genotypes <0.9, call rate <0.97, heterozygosity rate >4 standard deviations from the mean, discordance between originally provided and inferred genders or identity by descent $\pi > 0.9$ with another sample (the sample with lowest call rate was removed from each related pair). Variants were removed if they had low call rate (<0.99 for variants with minor allele frequency (MAF) <0.05 or <0.97 for variants with MAF≥0.05), significantly different missing rates between cases and controls ($p<1\times10^{-6}$) or significant deviation from Hardy-Weinberg equilibrium (HWE) (exact test $p<1\times10^{-6}$).

The AADM samples were genotyped on the Affymetrix Axiom® PANAFR SNP array as described previously [3]. Genotype calling was performed using the Affymetrix® Genotyping Console™ Software (GTC) and following the manufacturer's best practices guidelines. Only samples with call rate >= 0.95 and heterozygosity < 4SD from the mean were included. AADM samples were excluded if they were duplicated, sex-discordant or showed cryptic relatedness with other individuals [3]. SNPs were filtered if they had call rate ≤95, HWE exact test ($p<1\times10^{-6}$) and MAF<0.01[3].

Imputation was performed using a merged panel in which the 1000 Genomes phase 3 panel[4] (release 20130502) was combined with 2,298 African samples with sequence data from the African Genome Variation Project (AGVP)[2] and the Uganda 2,000 Genomes Project (UG2G) (http://www.ashg.org/2014meeting/abstracts/fulltext/f140122667.htm) following a comparison with the 1000 Genomes phase 3 panel alone[5]. In the combined Zulu sample, all variants passing QC were flipped to the positive strand of the reference genome (build 37). Variants overlapping those in the imputation panel with MAF>0.01 (but less than 0.4 for A/T

or G/C variants) and difference in frequency <0.2 between the genotyped variant and the imputation panel were prephased using SHAPEITv2 [6] (Oxford University, Oxford, UK www.well.ox.ac.uk/~gav/resources/snptest_v2.5.2_linux_x86_64_dynamic.tgz) and imputed with IMPUTE2 [7]. The AADM samples were imputed to the same merged panel using the Sanger imputation server (https://imputation.sanger.ac.uk/). Following imputation, an updated reference panel was released (the African Genome Resources (AGR) panel available on the Sanger imputation server). We retained all imputed SNPs also in the AGR panel with MAF>1% and imputation information score>0.4.

Meta-analysis, signal selection and fine-mapping

Meta-analysis of the Zulu and AADM summary statistics for shared variants was performed using a fixed-effects meta-analysis (weighted for effective sample size) in METAL [8]. Results for each cohort were corrected using the genomic control inflation factor, λ_{GC} [9]: Zulu λ_{GC} = 1.008, AADM λ_{GC} = 1.019. The meta-analysis results were further corrected for a second round of genomic control (λ_{GC} = 1.006). Meta-analysis odds ratios (OR) were estimated by performing a fixed-effect inverse variance meta-analysis in METAL, using an approximation of the Zulu allelic $\log_e$OR and variance from the allelic effect estimate from the mixed linear regression model [10, 11]. To identify distinct signals of association, we performed approximate conditional analyses using the joint model implemented in GCTA [12, 13]. LD was estimated from 2,959 African reference samples included in the merged panel. We included any variant with MAF>1% in both studies and applied the default values to other parameters of GCTA (p-value threshold = 1×10^{-5}). Finally, variants with $p < 2.5\text{x}10^{-8}$ in the joint model were selected as signals with genome-wide significance[14].

The Bayesian fine-mapping method FINEMAP [15] (Christian Benner, Helsinki, Finland www.christianbenner.com/finemap_v1.1_x86_64.tgz) was utilized to identify likely causal SNPs within 500kb either side of the most significant variant at each locus. Summary statistics (z-scores) from the sample size weighted meta-analysis of variants present in both Zulu and AADM and the pairwise Pearson correlations of variants from the African samples in the merged panel at each locus were supplied as input to FINEMAP. At each locus we ran FINEMAP assuming up to 5 causal variants (the default). We then iteratively ran FINEMAP setting the maximum number of causal variants to the number of causal variants supported by the Bayes' factor from the previous run until the number of causal variants converged. FINEMAP calculates the posterior probabilities of each variant being causal and proposes the most likely configurations of causal variants. We constructed 99% credible sets by including variants in the top configurations whose posterior probabilities summed to just over 99%. For comparison, we also performed the fine-mapping in each region using the approximate Bayes' factor approach [16, 17] with allelic effect estimates from the inverse variance meta-analysis assuming up to one causal variant without conditional analyses. We similarly performed finemapping in Europeans using the results from DIAGRAM [18] with 5,000 randomly selected samples from UK Biobank to estimate LD between variants.

Comparison with established loci

We used "direct" and "local" detection to explore the extent to which existing GWAS signals (almost all from non-African samples) were detected in the African GWAS. We used GARFIELD [19] to test for an enrichment of variants with p-value < 0.05 in the 100 previously established T2D signals (ESM Methods, ESM Table 2) compared to all other variants with $p<0.05$ in the African T2D meta-analysis. We generated annotation files for each variant in the African GWAS as input for GARFIELD: (i) genome-wide coordinates and

p-values for association with T2D from the African meta-analysis, (ii) African specific LD tag files (r2=0.1 and r2=0.8 within 1 Mb, estimated from the merged panel), (iii) minor allele frequencies from the African meta-analysis and distance to nearest transcription start site as supplied in GARFIELD (GRCh37) (iv) a file annotating each of the variants in the African meta-analysis as 1 if they were an established T2D variant (or their proxy, ESM Methods, ESM Table 2), and 0 otherwise.

In a "direct" comparison, we tested index variants at previously-reported T2D GWAS (taking $p<0.05$, and directional consistency, as evidence of detection in our data). At loci detected using this "direct" approach, we examined association profiles in the African data to ensure that the association at the index variant did not reflect LD with a stronger signal elsewhere in the region.

We also considered the evidence for "local" detection. These analyses aimed to detect locus-level replication which did not involve the same index variant (reflecting allelic heterogeneity or haplotypic diversity). For these "local" analyses, an association signal was deemed to have been replicated if at least one variant within the 200kb region flanking the previously-reported index variant (100kb either side) reached nominal significance threshold ($p<0.05$) after correcting for the effective number of independent tests (N_eff). N_eff was estimated using the software GEC as previously reported [20]*:* this test was well-calibrated in null data analyses (data not shown).

We performed genetic risk score (GRS) analyses to harvest association information from multiple variants. In both the AADM and Zulu samples, T2D association was tested by logistic regression adjusting for age, sex, and the first three principal components (PC) in unrelated individuals (in the Zulu samples, we performed an additional step to remove individuals with higher missing rate in each first-degree relative pair, 0.3). GRS were

calculated as the total number of risk alleles in subsets of the 102 variants at established loci from existing GWAS studies of T2D (published before February 2018), primarily in populations of European and Asian ancestry (ESM Table 2).

Testing association of *INS*-VNTR with type 2 diabetes

We used the haplotypic information for *INS*-VNTR generated in African-descent individuals by Stead *et. al*. (2003)[21] to impute *INS*-VNTR lineages in the Zulu and AADM samples. We used SHAPEITv2 [6] and IMPUTE2 [7] to phase and construct haplotypes and identify the *INS*-VNTR lineages and tested each for association with T2D. Of the 56 variants reported by Stead *et. al*. (2003) [21], 45 had positions in build 37 of which 43 were available in the Zulu and AADM samples. Each lineage was tested for association with T2D using a linear mixed model, implemented in GEMMA[22], adjusting for sex, age, and BMI (Zulu) or using a logistic model in R adjusting for age, sex, BMI and the first 3 PCs (AADM). Meta-analysis of the Zulu and AADM results was performed using an inverse variance fixed-effects meta-analysis (with an approximation of the allelic $\log_e$OR and variance from the linear model in the Zulu sample[10]). Conditional analysis was performed to detect distinct association signals by inclusion of dosages of the lead T2D variants as covariates in the regression model.

References

[1] Hird TR, Pirie FJ, Esterhuizen TM, et al. (2016) Burden of Diabetes and First Evidence for the Utility of HbA1c for Diagnosis and Detection of Diabetes in Urban Black South Africans: The Durban Diabetes Study. PloS one 11: e0161966

[2] Hird TR, Young EH, Pirie FJ, et al. (2016) Study profile: the Durban Diabetes Study (DDS): a platform for chronic disease research. Global Health, Epidemiology and Genomics 1

[3] Adeyemo AA, Tekola-Ayele F, Doumatey AP, et al. (2015) Evaluation of Genome Wide Association Study Associated Type 2 Diabetes Susceptibility Loci in Sub Saharan Africans. Front Genet 6: 335

[4] Genomes Project C, Auton A, Brooks LD, et al. (2015) A global reference for human genetic variation. Nature 526: 68-74

[5] (2016) Abstracts (abstract number 54). Genet Epidemiol 40: 609-674

[6] Delaneau O, Marchini J, Zagury JF (2011) A linear complexity phasing method for thousands of genomes. Nat Methods 9: 179-181

[7] Howie BN, Donnelly P, Marchini J (2009) A flexible and accurate genotype imputation method for the next generation of genome-wide association studies. PLoS Genet 5: e1000529

[8] Willer CJ, Li Y, Abecasis GR (2010) METAL: fast and efficient meta-analysis of genomewide association scans. Bioinformatics 26: 2190-2191

[9] Devlin B, Roeder K (1999) Genomic control for association studies. Biometrics 55: 997-1004

[10] Cook JP, Mahajan A, Morris AP (2017) Guidance for the utility of linear models in meta-analysis of genetic association studies of binary phenotypes. Eur J Hum Genet 25: 240-245

[11] Pirinen M, Donnelly P, Spencer CCA (2013) Efficient computation with a linear mixed model on large-scale data sets with applications to genetic studies: 369-390

[12] Yang J, Lee SH, Goddard ME, Visscher PM (2011) GCTA: a tool for genome-wide complex trait analysis. Am J Hum Genet 88: 76-82

[13] Yang J, Ferreira T, Morris AP, et al. (2012) Conditional and joint multiple-SNP analysis of GWAS summary statistics identifies additional variants influencing complex traits. Nature genetics 44: 369-375, S361-363

[14] Pe'er I, Yelensky R, Altshuler D, Daly MJ (2008) Estimation of the multiple testing burden for genomewide association studies of nearly all common variants. Genet Epidemiol 32: 381-385

[15] Benner C, Spencer CC, Havulinna AS, Salomaa V, Ripatti S, Pirinen M (2016) FINEMAP: efficient variable selection using summary data from genome-wide association studies. Bioinformatics 32: 1493-1501

[16] Wakefield J (2007) A Bayesian measure of the probability of false discovery in genetic epidemiology studies. Am J Hum Genet 81: 208-227

[17] Gaulton KJ, Ferreira T, Lee Y, et al. (2015) Genetic fine mapping and genomic annotation defines causal mechanisms at type 2 diabetes susceptibility loci. Nat Genet 47: 1415-1425

[18] Scott RA, Scott LJ, Magi R, et al. (2017) An Expanded Genome-Wide Association Study of Type 2 Diabetes in Europeans. Diabetes 66: 2888-2902

[19] Iotchkova V, Ritchie GRS, Geihs M, et al. (2019) GARFIELD classifies disease-relevant genomic features through integration of functional annotations with association signals. Nature genetics 51: 343-353
[20] Li MX, Yeung JM, Cherny SS, Sham PC (2012) Evaluating the effective numbers of independent tests and significant p-value thresholds in commercial genotyping arrays and public imputation reference datasets. Hum Genet 131: 747-756
[21] Stead JD, Hurles ME, Jeffreys AJ (2003) Global haplotype diversity in the human insulin gene region. Genome Res 13: 2101-2111
[22] Zhou X, Stephens M (2012) Genome-wide efficient mixed-model analysis for association studies. Nature genetics 44: 821-824
[23] Pruim RJ, Welch RP, Sanna S, et al. (2010) LocusZoom: regional visualization of genome-wide association scan results. Bioinformatics 26: 2336-2337

ESM Figures

ESM Fig. 1. Locuszoom plot of locus *TCF7L2* with fine-mapping configurations. The top panel shows the Locuszoom [23] plot of the 1Mb *TCF7L2* region spanning the lead SNP rs7903146. LocusZoom finds which of the reference SNPs (with symbols in red and blue) each variant in the region is in highest LD with, and colours each variant with a shade of red or blue to represent the LD between them and their matched reference SNP. The bottom panel shows the top five configurations from FINEMAP assuming there were up to five causal variants with the top configuration marked in yellow. The length of each rectangle represents the posterior probability of each configuration, shown on the left, and the rsIDs on the right are the variants within each configuration.

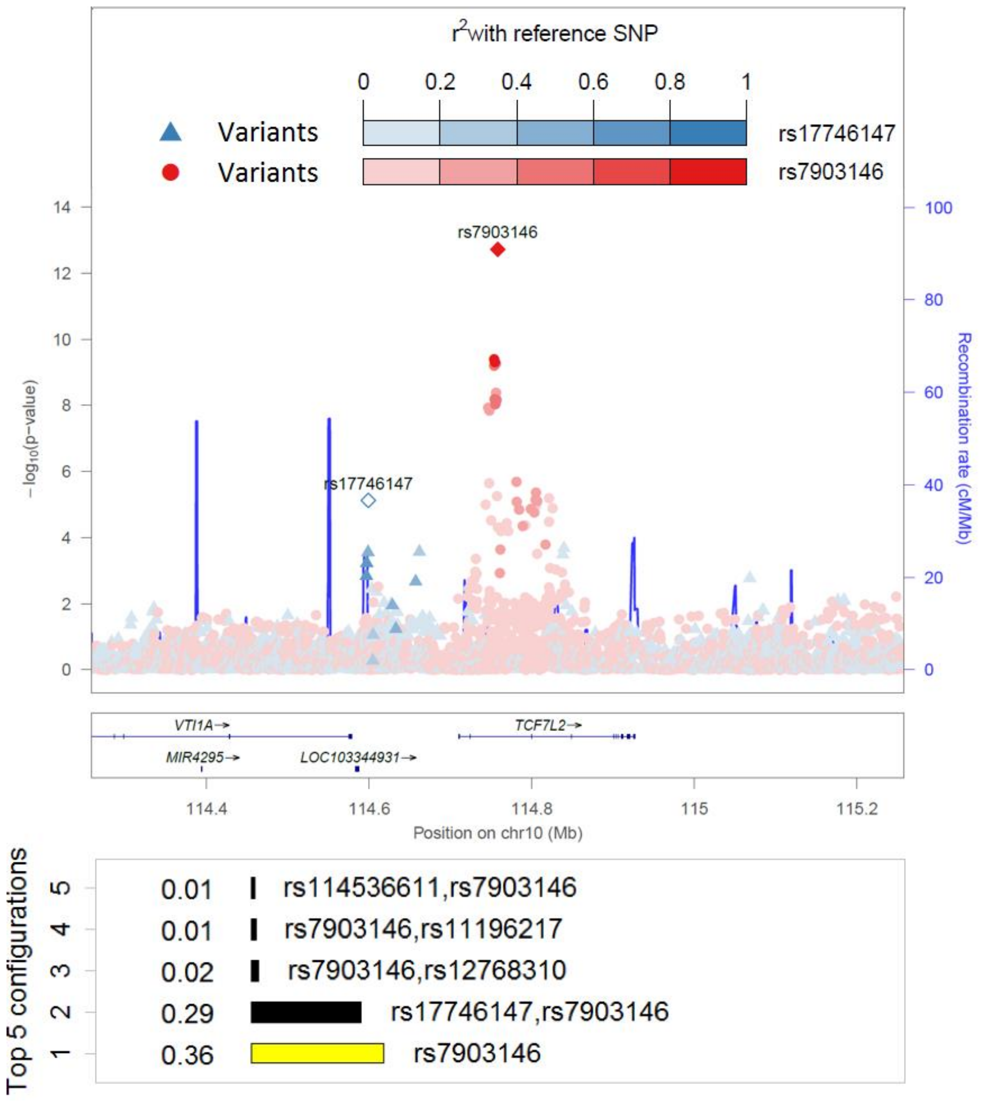

ESM Fig. 2. Locuszoom plot of locus *AGMO* with fine-mapping configurations. The top panel shows the Locuszoom [23] plot of the 1Mb *AGMO* region spanning the lead SNP rs73284431. LocusZoom finds which of the reference SNPs (with symbols in red and blue) each variant in the region is in highest LD with, and colours each variant with a shade of red or blue to represent the LD between them and their matched reference SNP. The bottom panel shows the top five configurations from FINEMAP assuming there were up to five causal variants with the top configuration marked in yellow. The length of each rectangle represents the posterior probability of each configuration, shown on the left, and the rsIDs on the right are the variants within each configuration.

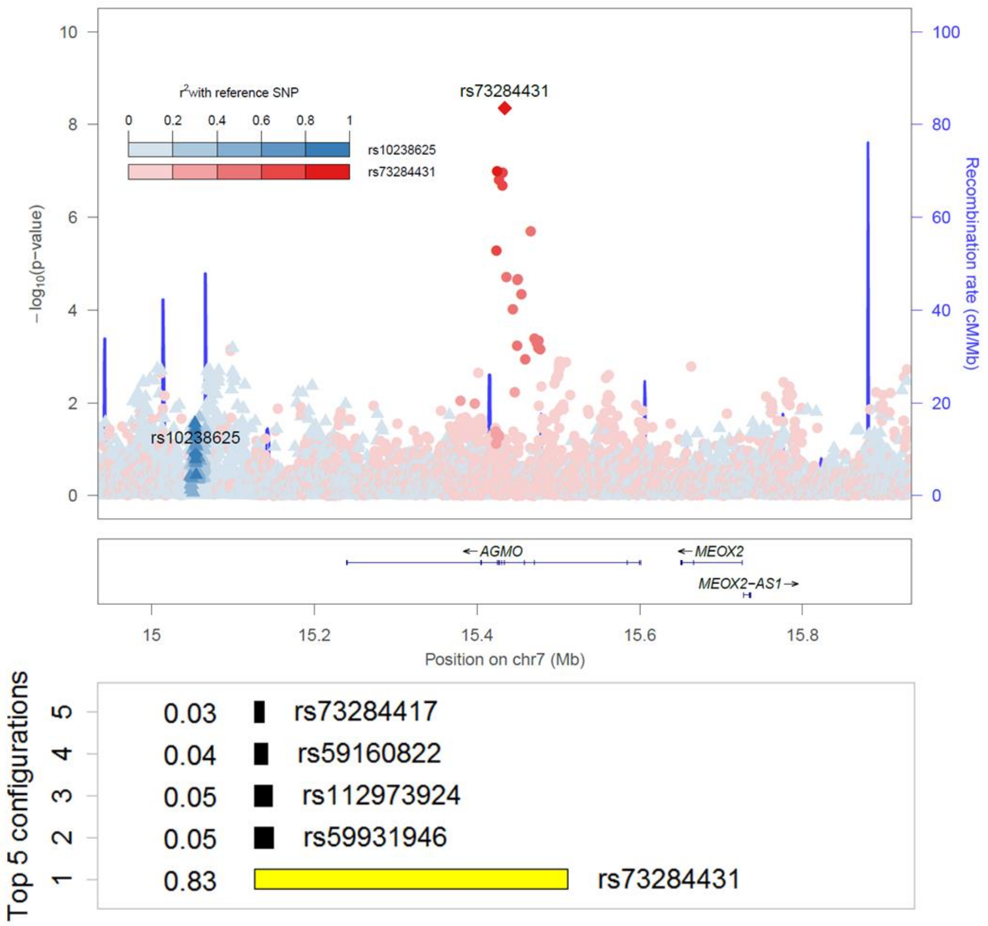

ESM Tables (see the separate file)

ESM Table 1. Sample characteristics of the Zulu and AADM samples included in the meta-analysis.

ESM Table 2. Direct and local detection of established loci.

ESM Table 3. T2D susceptibility loci with combined Zulu and AADM meta-analysis with $2.5x10^{-8} \leq$ p-value < $1x10^{-5}$

ESM Table 4. Lead variant at *AGMO* (rs73284431) conditional on previously reported variants (rs10238625, rs10276674).

ESM Table 5. T2D reciprocal conditional analysis results between the lead variants in Zulu/AADM meta-analysis (rs12277475) and reported index variants rs3842770, rs7107784 at the *INS* locus.

ESM Table 6. VNTR lineages and lineage groups associated with T2D in Zulu, AADM and combined (frequency in Zulu/AADM > 0.01).

ESM Table 7. T2D reciprocal conditional analysis results between VNTR lineage groups W and K, lead SNP rs12277475 and reported index variants rs3842770, rs7107784 in the Zulu data.

ESM Table 8. T2D reciprocal conditional analysis results between VNTR lineage groups W and P, lead SNP rs12277475 and reported index variants rs3842770, rs7107784 in AADM.

ESM Table 9. Finemapping of signals with posterior probabilities > 0.2 identified in this study in our African samples and comparison with Europeans.

ESM Table 10. Posterior probabilities of the five hypotheses given by "coloc" regarding whether previously reported T2D signals and African signals share the same causal variants in the 22 loci replicated by either the direct or the local approach.
